# Nanostructured 2D WS_2_@PANI nanohybrids for electrochemical energy storage

**DOI:** 10.3389/fchem.2022.1000910

**Published:** 2022-09-08

**Authors:** Matteo Crisci, Felix Boll, Leonardo Merola, Jonas Johannes Pflug, Zheming Liu, Jaime Gallego, Francesco Lamberti, Teresa Gatti

**Affiliations:** ^1^ Institute of Physical Chemistry, Justus Liebig University, Giessen, Germany; ^2^ Center for Materials Research, Justus Liebig University, Giessen, Germany; ^3^ Nanochemistry Department, Istituto Italiano di Tecnologia, Genova, Italy; ^4^ Department of Chemical Sciences, University of Padova, Padova, Italy

**Keywords:** 2D material, transition metal dichacogenide, nanohybrid, nanostructuring, energy storage

## Abstract

2D materials are interesting flat nanoplatforms for the implementation of different electrochemical processes, due to the high surface area and tunable electronic properties. 2D transition metal dichalcogenides (TMDs) can be produced through convenient top-down liquid-phase exfoliation (LPE) methods and present capacitive behaviour that can be exploited for energy storage applications. However, in their thermodynamically stable 2H crystalline phase, they present poor electrical conductivity, being this phase a purely semiconducting one. Combination with conducting polymers like polyaniline (PANI), into nanohybrids, can provide better properties for the scope. In this work, we report on the preparation of 2D WS_2_@PANI hybrid materials in which we exploit the LPE TMD nanoflakes as scaffolds, onto which induce the *in-situ* aniline polymerization and thus achieve porous architectures, with the help of surfactants and sodium chloride acting as templating agents. We characterize these species for their capacitive behaviour in neutral pH, achieving maximum specific capacitance of 160 F/g at a current density of 1 A/g, demonstrating the attractiveness of similar nanohybrids for future use in low-cost, easy-to-make supercapacitor devices.

## Introduction

Electrochemical energy storage is a central topic in current technology-oriented research (Zhang, 2013; Dutta et al., 2022) and the need to develop devices for different type of applications, ranging from automotive (Xu et al., 2020; Rajagopal et al., 2022) up to wearable electronics (Sumboja et al., 2018) and health monitoring platforms (Chen et al., 2020), triggers the continuous search for novel active materials that can satisfy these variable price and performance/durability demands. In this context, two-dimensional (2D) materials are attracting considerable attention, due to their high surface area and highly tunable electronic and electrochemical properties (Cui et al., 2020), as well as convenient methods for their large scale production. In particular, the top-down liquid-phase exfoliation (LPE) of bulk layered crystals (Hernandez et al., 2008; Nicolosi et al., 2013; Paton et al., 2014) is a very promising production method, allowing to obtain 2D nanosheets in colloidal form, i.e., as functional inks (Hu et al., 2018; Tian, 2021; Pinilla et al., 2022), suitable for the subsequent processing of solid-state architectures.

Among the many different 2D materials that have been produced and studied in the last decade, 2D transition metal dichalcogenides (TMDs) display interesting electronic and mechanical properties for use in energy storage systems (Cherusseri et al., 2019): in fact, the combination of large surface area and variable oxidation states opens up the possibility to store charges through both the electrical double layer and the pseudocapacitive mechanisms. Unfortunately, the most thermodynamically stable crystalline phase of TMDs, i.e., the 2H phase, is semiconducting, strongly limiting the charge/discharge potential achievable from these nanomaterials. The metastable metallic 1T phase can be alternatively produced through LPE by pre-treating the bulk crystalline material with organolithium compounds (Qian et al., 2020), but concerns about stability under prolonged operation in devices are still an open issue (Tang and Jiang, 2015; Jenjeti et al., 2021). An alternative to the use of bare 2D TMDs comes from the combination of these last ones with other (electro)active functional materials, into composite or nanohybrid (Osella et al., 2021; Versaci et al., 2022) structures. Since many years, conducting polymers represent a valuable option for this type of functional hybridization approach: they are low-cost and light-weight materials, they can be pre-synthesized or directly polymerized *in-situ* and they feature a wide range of convenient electronic properties, which can be exploited for energy conversion and storage applications (Sajedi-Moghaddam et al., 2017). Polyaniline (PANI) in particular, represents a sort of benchmark material for the construction of easy-to-make electrochemical energy storage platforms, due to its excellent environmental and thermal stability, coupled to the high electrical conductivity, typical of the emeraldine salt form, obtained when polymerization is carried out in acidic conditions (Beygisangchin et al., 2021).

In this work, we study nanohybrids of 2D WS_2_, prepared from LPE of bulk WS_2_ powder in environmentally friendly water/isopropanol mixtures (LPE WS_2_), and *in situ* polymerized PANI (defined from now on 2D WS_2_@PANI), for electrochemical energy storage. Similar composite materials have been studied by Jelinek and co-workers in a recent contribution, targeting the formation of microporous frameworks, in which efficient ion diffusion takes place, and revealing interesting performance in a symmetric supercapacitor architecture (De Adhikari et al., 2021). In order to obtain well-integrated and porous nanoarchitectures, we here adopt a templating strategy that makes use of two different ionic surfactants, namely sodium dodecyl sulphate (SDS) and sodium cholate (NaCh), to promote the effective mixing of the 2D nanosheets and the conducting polymer and of sodium chloride, to tune the morphology and nanostructure of the hybrids, following an example already reported for pure PANI electrodes by Anbalagan and Sawant (2016). The choice of these two specific surfactants is based on their well-known and largely studied ability to efficiently disperse in water-based environments many low-dimensional materials and, in particular, TMDs, as thoroughly discussed in a recent, very comprehensive review on the topic (Hu et al., 2021). The thus obtained different hybrid species are characterized for their structural and electrochemical performance, revealing important structure-to-property-to-function relationships which can be exploited to identify the most promising combinations for use in supercapacitor-like devices.

## Experimental section

### Materials and methods

All chemicals and solvents were purchased from Sigma-Aldrich and used without any further purification. Raman spectra were recorded on a Bruker Senterra instrument using a 514 nm laser excitation source, a ×50 magnification lens, 2 mW laser power, a 5 s integration time and 20 co-additions. The samples were analysed in solution (LPE WS_2_ and LPE WS_2_ re-dispersed) or prepared by disposing powders over a silicon slide (Bulk WS_2_ and filtered LPE WS_2_) and then analysed. Powder X-ray diffraction (P-XRD) measurements were performed on PANanalytical B.V. Empyrean in the 5–75° range using a measurement step of 0.013° and hold time of 75 s. Transmission electron microscopy (TEM) was carried out on a JEOL-1100 transmission electron microscope with an acceleration voltage of 100 kV. The sample was prepared by dropping dilute suspensions of LPE WS_2_ onto carbon film-coated 200 mesh copper grids. Scanning electron microscopy (SEM) was performed on a Zeiss Merlin instrument at a working potential of 4 kV. Energy-dispersive x-ray analysis (EDX) was performed on the same instrument at a working potential of 8 kV, an electron beam current of 8 nA and a X-Max 50 Silicon Drift Detector with 50 mm^2^ active area and polymer window was used. Nitrogen physisorption experiments were performed at 77 K with an automated gas adsorption station Quadrasorb EVO by Quantachrome Instruments. Prior to the measurements, the samples were degassed in vacuum for 12 h at 50°C. The surface area was determined using the BET method, and the pore size distributions were calculated *via* the nonlocal density functional theory (NLDFT) approach, using the adsorption branch of the isotherm. X-ray photoelectron spectroscopy (XPS) measurements were conducted with a PHI 5000 VersaProbe II Scanning ESCA Microprobe (Physical Electronics) with monochromatized Al Kα X-ray source in high power mode (beam size 1,300 μm × 100 μm, X-ray power: 100 W). Time steps of 50 ms, a step size of 0.2 eV and an analyzer pass energy of 46.95 eV were used for measuring the detail spectra. The sample surface was charge neutralized with slow electrons and argon ions, and the pressure was in the range from 10^–7^ to 10^–6^ Pa during the measurement. Data analysis was performed using the CasaXPS software.

### Liquid-phase exfoliation of WS_2_

For LPE, WS_2_ powder with a particle size of 2 μm and a purity of 99% and sodium cholate hydrate (NaCh, from bovine and/or ovine bile) with a purity of 99% were employed. Up to a volume of 500 ml, LPE was carried out in a mixture of isopropanol and demineralized water at a ratio of 7:3 v/v. First, 1 mg/ml of NaCh was added to the solvent mixture and stirred until a clear solution was obtained. Afterwards, 10 mg/ml of the bulk WS_2_ powder was added to the solution under continuous stirring for 10 min. The suspension was then further homogenized for 30 min in an ultrasonic bath. The LPE was carried out employing an IKA T25 digital Ultra-Turrax shear mixer at 8,000 RPM for 4 h, while the suspension was covered and cooled in an ice bath to avoid solvent evaporation. The final mixture was allowed to undergo gravitational sedimentation for 4 days, after which the colour changed from black to a yellow-brown nuance in the supernatant, which was thus separated from the precipitate (pellet). This final LPE WS_2_ ink was filtered on an Omnipore-Teflon-Membrane (0.2 μm, from Merck Millipore), washed thoroughly on the filter with water to remove any NaCh residue and dried in vacuum, to determine the yield in 2D WS_2_ (∼2%). The LPE WS_2_ was then recovered from the filter by re-dispersing it in isopropanol with the help of the ultrasonic bath and isolated as a powder after isopropanol evaporation under reduced pressure.

### Synthesis of 2D WS_2_@PANI hybrids

Pure PANI was synthesized through oxidative polymerization by first preparing a 0.1 M solution of aniline in 1 M HCl saturated with NaCl (brine). In another container, a 1.15 M solution of K_2_S_2_O_8_ was prepared in 1 M HCl (also saturated with NaCl). Both solutions were then combined together and cooled to 0°C using an ice bath. After stirring for 30 min, the suspension was filtrated under vacuum and washed with 200 ml of 1 M HCl and twice with 25 ml of acetone. The formed product was dried at 40°C in a vacuum oven overnight. 0.4893 g of a dark green, almost black solid was obtained, which corresponds to a yield of 98%. For synthesis of the 2D WS_2_@PANI hybrids, a slightly modified procedure was used: first the surfactant (1.25 mg/ml for SDS, 1.5 mg/ml for NaCh) was dissolved in 1 M HCl saturated with brine. Afterwards the respective amount of the LPE WS_2_ powder (0.01, 0.02 and 0.05 equivalents compared to the used aniline) was added and dispersed by sonicating in an ultrasound bath at 37 Hz, 60% power for 15 min. Then, 0.245 ml of aniline were added to still form a 0.1 M solution. Afterwards, the mixture was combined with the same oxidant solution, as previously described, and the protocol continued as above. Since different WS_2_/PANI molar ratio were prepared, Table 1 summarizes the relative quantities for each involved chemical and the quantity of product obtained.

**TABLE 1:** Relative quantities (mg, mmol) for each involved reagent in the preparation of 2D WS_2_@PANI nanohybrids and the quantity of product obtained.

WS_2_/PANI molar ratio	K_2_S_2_O_8_ (mg, mmol)	Surfactant (mg, mmol)	LPE WS_2_ (mg, mmol)	Product (mg)
1:20 (+SDS)	919, 9.9	66, 0.2	35, 0.14	281
1:20 (+NaCh)	919, 9.9	77, 0.18	35, 0.14	370
1:50 (+SDS)	919, 9.9	66, 0.2	14, 0.06	331
1:50 (+NaCh)	919, 9.9	77, 0.18	14, 0.06	165
1:100 (+SDS)	919, 9.9	66, 0.2	7, 0.03	284
1:100 (+NaCh)	919, 9.9	77, 0.18	7, 0.03	345

### Electrochemical testing

The electrochemical performance of the 2D WS_2_@PANI hybrids was characterized by galvanostatic charge-discharge (GCD) and electrochemical impedance spectroscopy (EIS) using a three-electrode set up based on a glassy carbon electrode (GC) as working electrode (WE), a platinum wire as counter electrode (CE) and an Ag/AgCl reference electrode (RE). The data have been then translated against the reversible hydrogen electrode (RHE), for the sake of clarity. The tests were performed on an Autolab PGSTAT302 equipped with the Nova (2.1.1.) software. The WE was prepared as follows: an ink of 5 mg/ml of active material was produced by weighting the desired hybrid and dispersing it in 2 ml of H_2_O/EtOH (1:1 v/v). Afterwards 60 µl of Nafion (30 μl/ml of ink) were added to the suspension and the final ink was sonicated in ultrasonic bath (40% power, room temperature, 37 kHz frequency) for 1 h. Afterwards, 5 µl of the resulting ink were drop casted on top of the GC WE and dried in oven at 100°C for 1 h before use. The measurements were performed in 0.5 M Na_2_SO_4_ electrolyte. GCD measurements were performed in the voltage range 0–0.8 V at the different current densities of 0.2, 0.5, 1, 2, 5, 10 A/g. Based on the GCD curve, specific capacitance (C_S_) for the single electrode material was calculated using the equation:

$$C_{s}=\frac{I*\Delta t}{m*\;\Delta V}$$ (1)

where *I* is the discharge current applied, Δ*t* is the discharge time of the curve, *m* is the mass of active material on the electrode and Δ*V* is the potential window. EIS measurements were carried out on a BioLogic SP 200 potentiostat at a constant potential mode of 0 V. Frequency range was varied between 200 kHz and 10 Hz at an amplitude of 5 mV.

## Results and discussion

For the production of LPE WS_2_, we have optimized a protocol that resorts to the use of shear mixing in green isopropanol/water solvent mixtures (Crisci et al., 2022) in the presence of NaCh as surfactant, to assist the mechanical exfoliation by properly adjusting the liquid medium surface tension. Optimization has mostly been addressed at reducing as much as possible the amount of surfactant, while still obtaining a good yield in exfoliated 2D material ink, as well as to be able to scale-up the production up to 500 ml per batch. Remarkably, this process is providing yields that are similar to those obtained by resorting to more classical LPE in high boiling solvents like N-methylpyrrolidone (NMP), while being undoubtedly more suitable from an environmental perspective for the future industrialization of similar inks (Hu et al., 2021). We have then proceeded through a filtration, accompanied by thorough washings, to remove all the surfactant and obtain a powder of the LPE WS_2_, directly usable for the preparation of the 2D WS_2_@PANI nanohybrids. Although NaCh is also employed within this last process, we had the need to remove the native one, being interested also in testing another surfactant, namely SDS, to compare the effect of the two different molecular structures on the quality of the 2D WS_2_@PANI species.

In order to rule out the extensive re-aggregation of the individual few-layers WS_2_ nanosheets during filtration and to demonstrate the reversibility of such aggregation following a simple re-dispersion step in ultrasonic bath, we have carried out a thorough Raman analysis, following the detailed description reported in the work of Terrones and coworkers (Berkdemir et al., 2013). The results of this analysis are displayed in Supplementary Figure S1 of the Supplementary Material (SM). By examining the position and relative intensity of the 2LA + E^1^2g peak in the range 350–375 cm^−1^ and of the A_1g_ peak in the range 410–430 cm^−1^, it is possible to estimate the average number of layers present in a given WS_2_ sample. In Supplementary Figure S1, we show the spectra of the starting bulk WS_2_ in powder compared to that of the LPE WS_2_ ink in liquid, of the filtered LPE WS_2_ ink (as powder) and of the re-dispersed nanomaterial in 1 M HCl in brine in the presence of freshly added surfactant (as a suspension). In these spectra, the two Raman modes under investigation change significantly in position when going from the bulk to the LPE material (the 2LA + E^1^2g mode shifts from 348 to 364 cm^−1^ and the A_1g_ mode from 413 to 433 cm^−1^). Also in the filtered material, they never return to the original bulk position, although a slight shift to lower wavenumbers is detectable after filtration. The average number of layers in the nanosheets can be quantified by considering the ratio between the intensities of the 2LA + E^1^2g mode and the A_1g_ mode. This ratio is lower than 1 (0.95) in bulk WS_2_ and increases to a value higher than 2 (2.08) in the LPE WS_2_, indicating the production of nanosheets with an average number of layers between 1 and 3, thus a highly exfoliated nanomaterial. TEM images of this material (Supplementary Figure S2) demonstrate their few-layers nature (possibly some mono, bi and tri-layer species are distinguishable), although strong aggregation at the solid-state (i.e., in this case on the TEM grid) takes place, making difficult to distinguish individual flakes. In the filtered species, we observe a decrease of the ratio to values similar to the bulk (0.95), which anyway reconverts to 1.6 in the re-dispersed powder, typical of 2–5 layers nanosheets (Berkdemir et al., 2013), thus again mostly 2D layered species.

**FIGURE 1:**
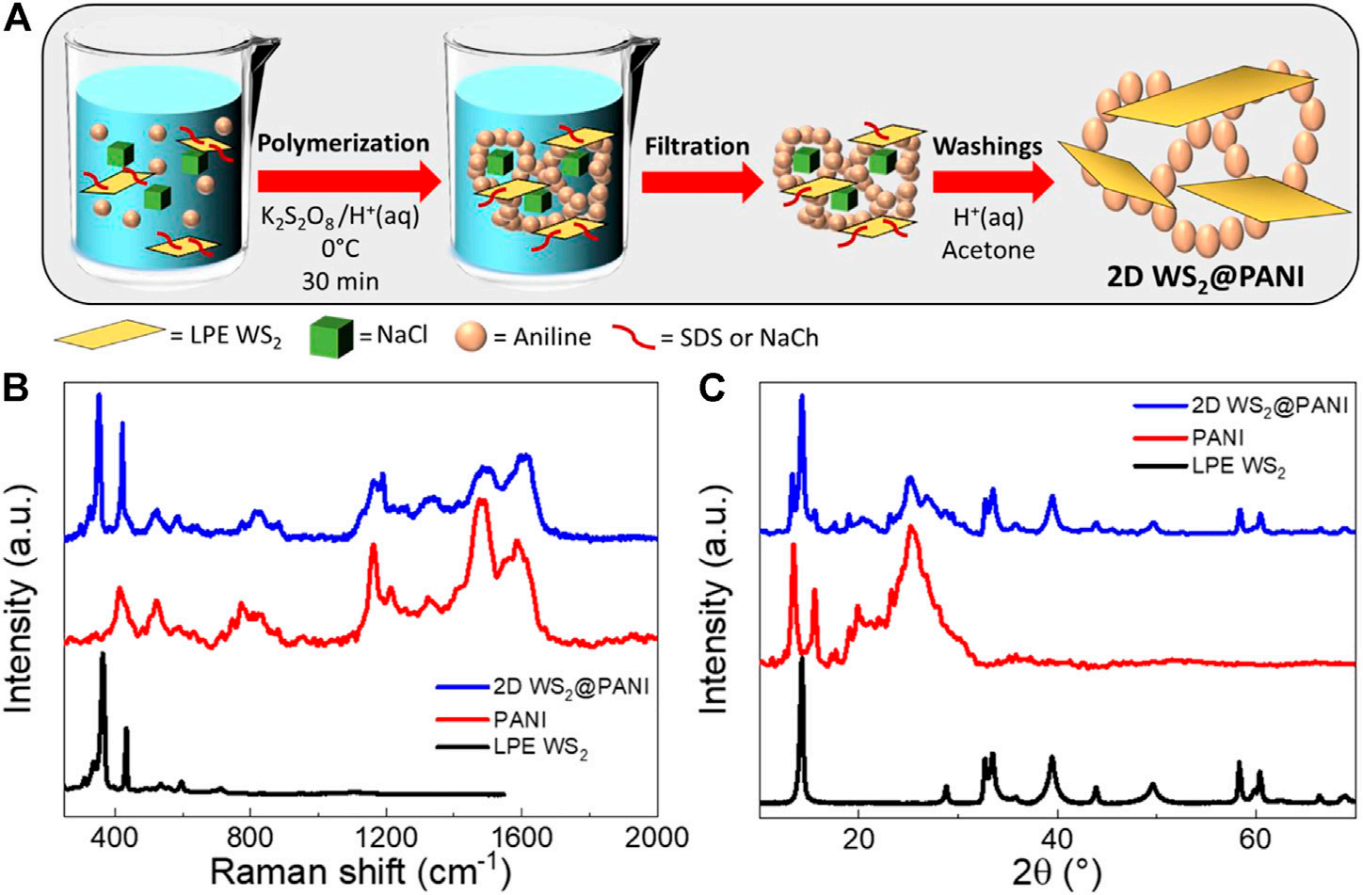
**(A)** Schematic representation of the template-assisted synthesis of nanostructured 2D WS_2_@PANI nanohybrids. **(B)** Raman spectra and **(C)** P-XRD diffractograms of the nanostructured 2D WS_2_@PANI nanohybrids, of template-assisted synthesized pure PANI and of LPE WS_2_ (the P-XRD pattern of this last one has been obtained from the filtered powder).

The procedure adopted to prepare nanostructured 2D WS_2_@PANI hybrids is depicted in Figure 1A, in which the effective mixing between the nanosheets and aniline is ensured by the presence of one of the two investigated surfactants (SDS or NaCh) and the formation of porous architectures is templated by the concomitant action of NaCl crystals (Anbalagan and Sawant, 2016) during the *in situ* oxidative polymerization, triggered by potassium persulfate used as the oxidant. The trapped NaCl crystals are then removed after thorough washings of the filtered composite materials, leaving behind the target 2D WS_2_@PANI hybrids. Other than varying the surfactant, we have also tuned the relative molar ratio between LPE WS_2_ and aniline, to produce hybrids with different stoichiometries, ranging from 1:20 to 1:50 and up to 1:100.

All the samples have been investigated through Raman and P-XRD analysis, of which prototypical spectra and diffractograms are reported in Figures 1B,C, respectively. The results are compared with the Raman spectra and P-XRD patterns of the two individual components, namely LPE WS_2_ and pure PANI, also prepared with the NaCl templating method. From Raman, we can distinguish in the nanohybrid the presence of the two previously discussed 2LA + E^1^2g and A^1g^ modes of layered WS_2_, with positions unvaried with respect to LPE WS_2_ and intensity ratio typical of highly exfoliated material, although not precisely determinable due to overlapping of the A_1g_ mode with a vibration of PANI (out of plane deformation of the ring structure). Concerning the PANI component in the hybrids, we can observe a change in the relative intensities of the two signals at around 1,600 cm^−1^ (stretching of the conjugated C-C bonds in the quinoid form) and at around 1,480 cm^−1^ (stretching of the C=N bond) with respect to pure PANI, likely indicating a change in the supramolecular structure of the polymeric/oligomeric chains, although extremely complex to exactly determine (Trchová et al., 2014; Stejskal et al., 2017). P-XRD also confirms the presence of the two crystalline components in the hybrids, although no particular additional information can be inferred, if not that residual NaCl is not present in the sample at detectable levels (the typical intense reflex of this last one at 2θ = 31.7° is absent in the diffractograms).

The templating and nano-structuring effect of the particular synthetic method here employed is well recognizable from the SEM characterization of the resulting nanohybrid materials. First of all, it should be pointed out that the mixing effect of the surfactant is essential to not achieve phase separation during the *in situ* aniline polymerization in the presence of the LPE WS_2_ flakes, as it is testified by the SEM image reported in Supplementary Figure S3 (right) of the SM. This was recorded on a 1:20 2D WS_2_@PANI sample, for which individual and aggregated 2D material flakes emerge within a background of the sole polymer scaffold (a SEM detail of the nanomorphology of pure PANI is also provided in the same figure, left, for the sake of comparison). The tendency to self-aggregate is indeed typical of the LPE WS_2_ material when casted from the liquid phase onto substrates, as it can be seen from the SEM image also reported in Supplementary Figure S3 (bottom image).

**FIGURE 2:**
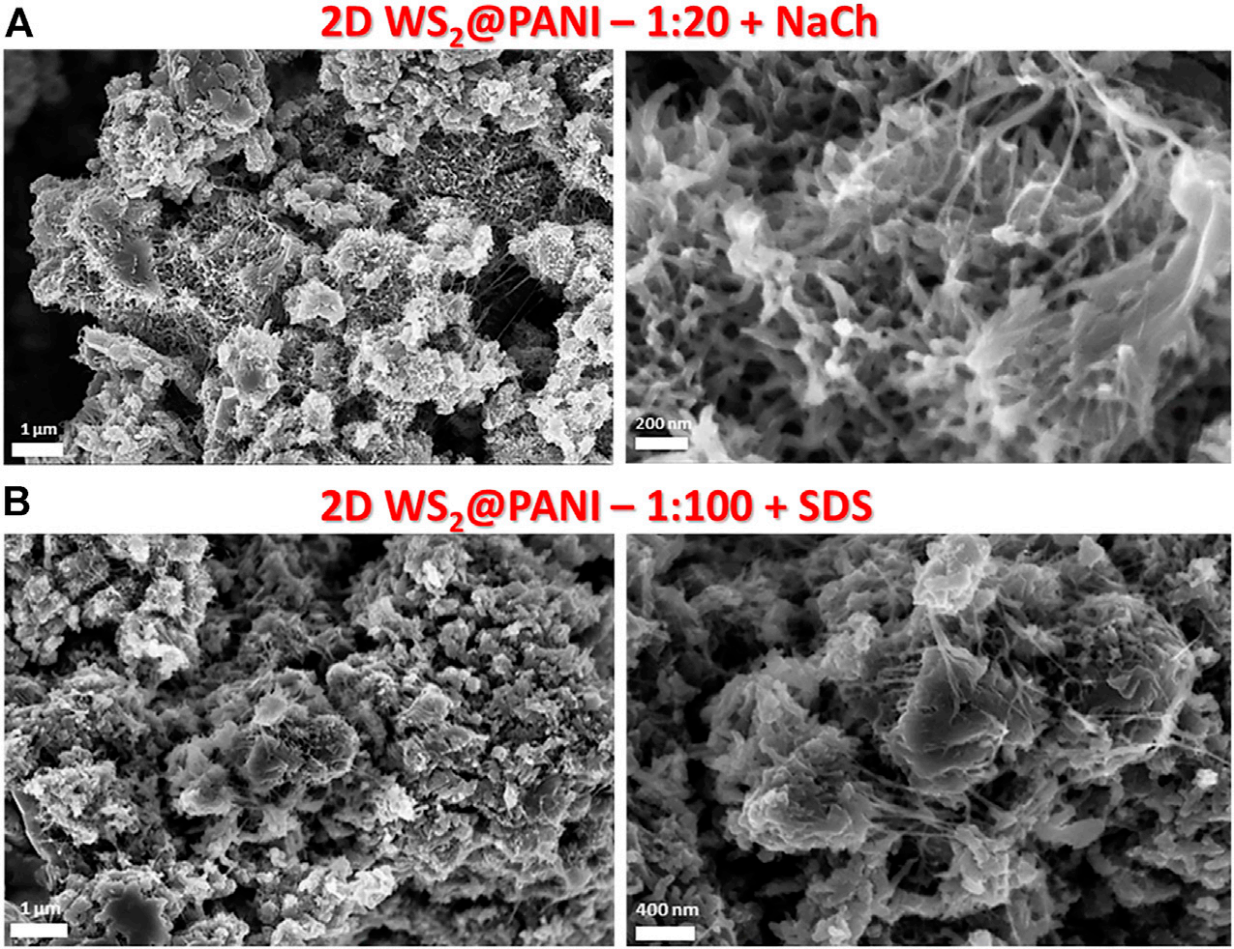
SEM images at different magnifications of prototypical nanostructured 2D WS_2_@PANI nanohybrids obtained with different LPE WS_2_/PANI molar ratios and with different surfactants favouring the proper mixing of the two individual components in the composite. **(A)** 2D WS_2_@PANI with 1:20 molar ratio prepared in the presence of NaCh as surfactant. **(B)** 2D WS_2_@PANI with 1:100 molar ratio prepared in the presence of SDS as surfactant.

Then, what clearly emerges from SEM images reported in Figure 2 and in Supplementary Figures S4, S5, is a certain difference in the nanomorphology of the different hybrids, obtained in the presence of either NaCh or SDS as the compatibilizer. In particular, for the NaCh-based 2D WS_2_@PANI hybrids we can distinguish already at the lowest 2D WS_2_/PANI ratio (1:20, Figure 2A) the formation of fractal-like nanostructures, most likely due to the NaCl-templated growth of PANI on the surface of the 2D WS_2_ nanosheets, coupled to filaments of the same polymer connecting different areas. These peculiar morphological characteristics are further maintained in the samples with higher 2D material/polymer ratios (Supplementary Figure S4). On the contrary, in the hybrids synthesized in the presence of SDS, the formation of similar complex nano-architectures is only detectable within the 1:100 2D WS_2_@PANI sample (Figure 2B), while at the higher TMD contents, the presence of aggregated TMD flakes separated from PANI aggregates is distinguishable. These data might indicate a different ability of the two surfactants to act as proper compatibilizers between the two materials in the composites. In particular, the more extended molecular structure of NaCh, compared to the mostly linear one of SDS, seems to better provide efficient intermixing between the 2D WS_2_ nanosheets and the conjugated polymer. Further studies are anyway necessary to better understand this behaviour. In addition, it is worthy to specify that the formation of the peculiar filament-like nanostructures in these composites is most likely attributed to the use of the saturated brine solution during the synthesis. During this process, NaCl crystallizes from the saturated solution and then re-dissolves during the washings, allowing the formation of short chains and filaments of PANI between the different polymeric domains.

**FIGURE 3:**
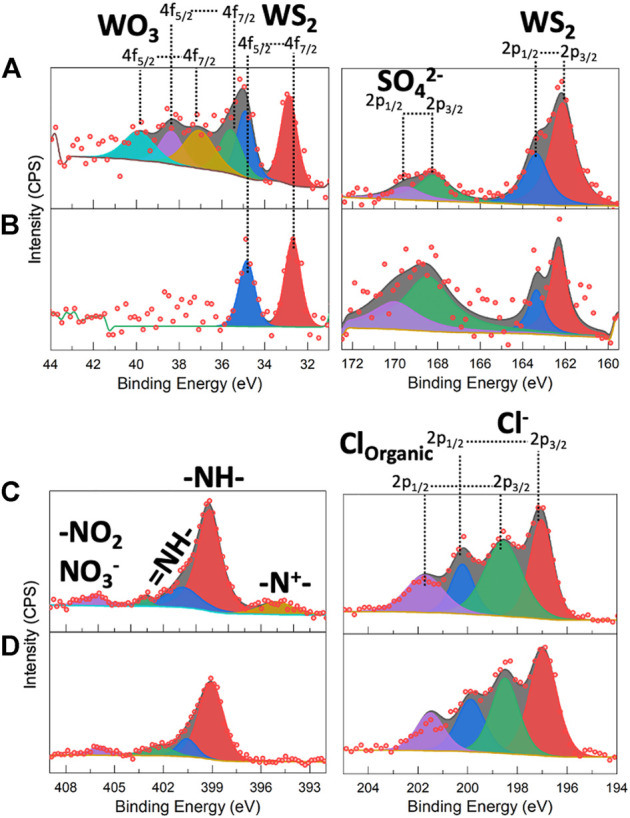
XPS spectra of **(A,C)** 2D WS_2_@PANI with 1:20 molar ratio prepared in the presence of SDS and **(B,D)** 2D WS_2_@PANI with 1:20 molar ratio prepared in the presence of NaCh in the W 4f, S 2p (top panels) and N 1s and Cl 2p (bottom panels) regions.

Although the degree of nano-structuring in the synthesized hybrids seems to point out at different overall surface areas for the various type of samples, physisorption data were not varying significantly across both the NaCh- and SDS-based series. Type III N_2_ physisorption isotherms were found for all the investigated species (a prototypical one is reported in Supplementary Figure S6), indicating the presence of mostly macropores and surface areas from Brunauer–Emmett–Teller (BET) analysis all in the range 23–27 m^2^/g, thus considerably lower than what found for similar species recently (up 70 m^2^/g in the work of De Adhikari et al. (2021). We completed the compositional analysis of the 2D WS_2_@PANI hybrids by comparing XPS and SEM-EDX data for the 1:20 samples prepared in the presence of both SDS and NaCh as compatibilizers. XPS spectra of the tungsten, sulphur, nitrogen and chlorine regions of these species are reported in Figure 3, while the entire spectra are displayed in Supplementary Figure S6, for the sake of completeness. From the W 4f region of the SDS-based hybrid, it is clearly distinguishable the presence of tungsten oxide species together with the native sulphide (Figure 3A). As the oxide is not detectable from P-XRD, it must be related to the sole surface, coming from the there-happening partial oxidation of WS_2_. These oxidized species are not detected in the XPS spectrum of the NaCh-based hybrid (Figure 3B), in which only the signals of WS_2_ are present: we can therefore argue that the PANI nanostructures seen in the SEM images of the latter and not in those of the former (at least not in the 1:20 sample), act as an effective protecting layer against the surface oxidation of the TMD nanosheets. The S 2p region shows in both samples the presence of WS_2_ and of sulfate species, which are most likely related to residues of the oxidant used for PANI polymerization. From N 1s region, it is detected the PANI component in the hybrids, characterized by mostly -NH- and = NH- groups along the chains coming from the aromatic and quinoid polymer forms, but also from small cationic and oxidized nitrogen species, particularly in the SDS-based sample. The level of PANI p-doping can be inferred by analysing the Cl 2p region, where both chloride ions and organic chloride (i.e., chloride directly bonded to carbon atoms) can be distinguished (still, uncertainty would remain on whether some of the chloride signals are not originating from not perfectly washed away NaCl used during the synthesis, although no sodium signal is seen in the XPS spectra, Supplementary Figure S7, indicating the lack of this element on the surface). Qualitatively, the spectra of the two species are not differing significantly, but it is possible to quantify the atomic percentage of the various elements and calculate from that the Cl/N ratio, which is directly related to the extent of doping, since chlorides act as counter-ions for the doped form of the polymer.

In Table 2, we show this quantitative data for the 1:20 SDS- and NaCh-based 2D WS_2_@PANI hybrids, calculated from both XPS and SEM-EDX analysis, with the former providing information on the surface composition while the latter of the bulk. As it can be inferred from this numbers, the Cl/N ratio is slightly higher in the NaCh-based sample, although here no clear signal of −N^+^- species (that are counterions of Cl^−^ in the p-doped PANI) are present in the N 1s region (Figure 3C).

**TABLE 2:** Comparison between atomic % of different elements in 2D WS2@PANI nanohybrids as obtained from XPS and EDX analysis.

At. %	C	N	O	Cl	W	S	S/W ratio	Cl/N ratio
2D-WS2@PANI 1:20 + SDS								
XPS	76.4	11.3	6.1	4.1	0.15	2.05	13.6	0.4
EDX	82.5	10.5	1.1	3.5	0.3	0.4	1.33	0.33
2D-WS2@PANI 1:20 + NaCh								
XPS	74.7	7.8	7.5	4	0.1	0.8	12	0.5
EDX	85.2	10.7	0.8	1.3	0.8	1.2	1.5	0.12

The S/W ratio could provide some information on the stoichiometry of the WS2 component, although this number is strongly influenced by the presence of the oxidant residues (SO_4_^2−^) and of the sulphate polar head in SDS molecules. Indeed, this ratio is considerably high on the surface, where mot likely these sulphur-containing species are prevalently located, while in the bulk it is closed to the expected value (2), with a slight under-stoichiometric tendency that might be related to sulphur-vacancies present on the 2D WS2 nanosheets (most likely on the edges). Supplementary Figure S8 reports the SEM-EDX maps of the different analysed elements in the 1:20 2D WS2@PANI hybrids prepared in the presence of SDS and NaCh. Here, we can notice the inhomogeneous nature of the former composite, characterized by areas in which WS2 seems confined (W and S signals distribution), while the PANI component is present everywhere (N, C and Cl signals). These data further reinforce the previous speculation made from simple observation of the SEM micrographs, i.e., the low extent of intimate mixing between the two materials in the SDS-containing 1:20 2D WS2@PANI hybrid. On the other hand, for the NaCh-containing hybrid, we can further confirm from Supplementary Figure S7 the highly homogenous nature, with the W and S signals extending over a large part of the mapped area as are those of N and C.

**FIGURE 4:**
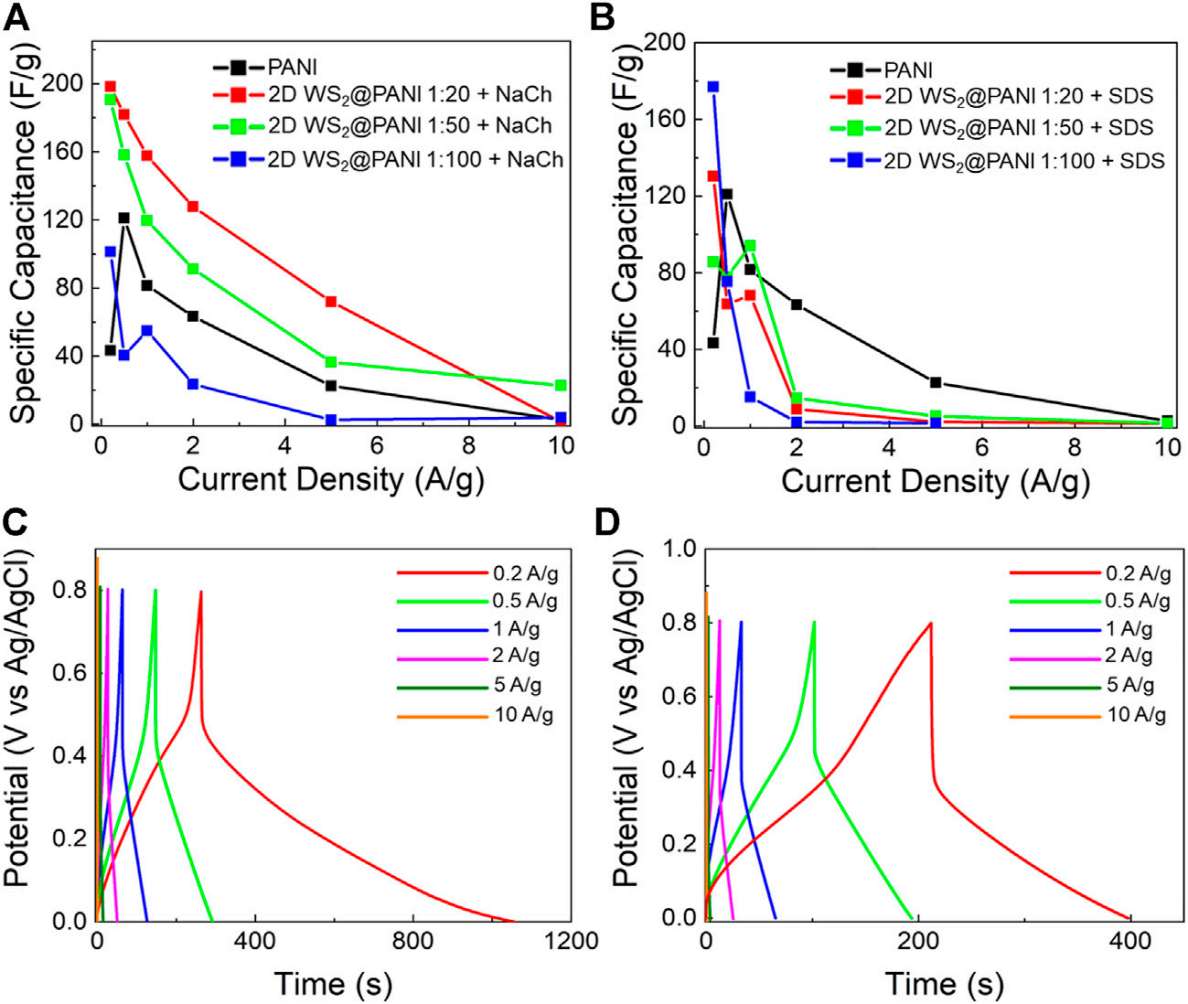
Electrochemical performance of 2D WS2@PANI nanohybrids at neutral pH (in 0.5 M Na2SO4 electrolyte). Specific capacitance as a function of current density for 2D WS2@PANI nanohybrids at different molar ratios prepared in the presence of **(A)** NaCh and **(B)** SDS. The data for pure PANI are also provided for the sake of comparison. GCD curves at increasing current densities for **(C)** the 1:20 2D WS2@PANI nanohybrid prepared in the presence of NaCh and **(D)** for pure PANI.

The series of synthesized 2D WS2@PANI hybrids has been characterized for its electrochemical performance in light of the charge storage capability at neutral pH (Figure 4), by investigating them through GCD. The materials have been displaced in the form of powder samples on a GC electrode with the help of Nafion as binder to ensure their safe and stable attachment. The choice of operating at neutral pH is specific for the perspective of employing them in wearable electronics, although it is well known that PANI-based species demonstrate higher capacitances in strongly acidic media (Li et al., 2009).

Figures 4A,B display the calculated CS from GCD curves (Supplementary Figure S9, Figures 4C,D) as a function of the current density for the composites prepared in the presence of, respectively, NaCh and SDS at the different 2D WS2/PANI ratios.

The performance of the two NaCh-containing samples featuring the lowest amount of PANI (1:20 and 1:50) is rather similar and always surpassing that of pure PANI up to high current densities. Remarkably, the CS value at 1 A/g of the 1:20 sample is 160 F/g, thus almost doubling that of pure PANI. In addition, this value is also slightly higher than the CS value at such current density reported by De Adhikari et al. (2021) from similar hybrid materials (140 F/@ 1 A/g) featuring even higher surface areas, pointing out at the existence of other effects governing the capacitive behaviour in these systems. However, the same cannot be observed for the SDS-based samples, which overall have worst performances than the pristine PANI reference, with the exception of the extremely low current densities regime. The present data seem to confirm the generally lower quality of the constituent materials mutual mixing and nano/microstructure in the SDS-based composites in comparison with the NaCh-based ones, which translates in a poorer electrochemical charge storage ability. The WS2 surface oxidation detected by XPS in these systems, might also be a partial cause of their poor performance, as generally the oxide is less conductive than the sulphide (Liu et al., 2015). Since the capacitive behaviour of pure 2D WS2 is almost negligible (and for this reason not shown here), the results obtained for the 1:20 2D WS2@PANI hybrid prepared in presence of NaCh point out at a notable cooperative effect between the TMD nanosheets and the conducting polymer, which allows to overperform both the individual species involved. This is even more evident when considering that by increasing the conducting polymer content, and thus going more in the direction of a mostly polymer-based active material, the energy storage capability worsens progressively and becomes even lower than that of pure PANI (in the 1:100 sample).

To better characterize the electrochemical behaviour of the champion composite (i.e., the 1:20 2D WS2@PANI + NaCh), we examined more in detail the GCD curves of this material at increasing current densities (Figure 4C). The GCD curves allow to appreciate the longer discharge time typical of the hybrid in comparison to the pure PANI reference (Figure 4D), which contribute to the higher CS measured for the former. In addition, their asymmetric shape is a sign of the pseudocapacitive behaviour of these materials over the range of current densities examined (Chen et al., 2013).

**FIGURE 5:**
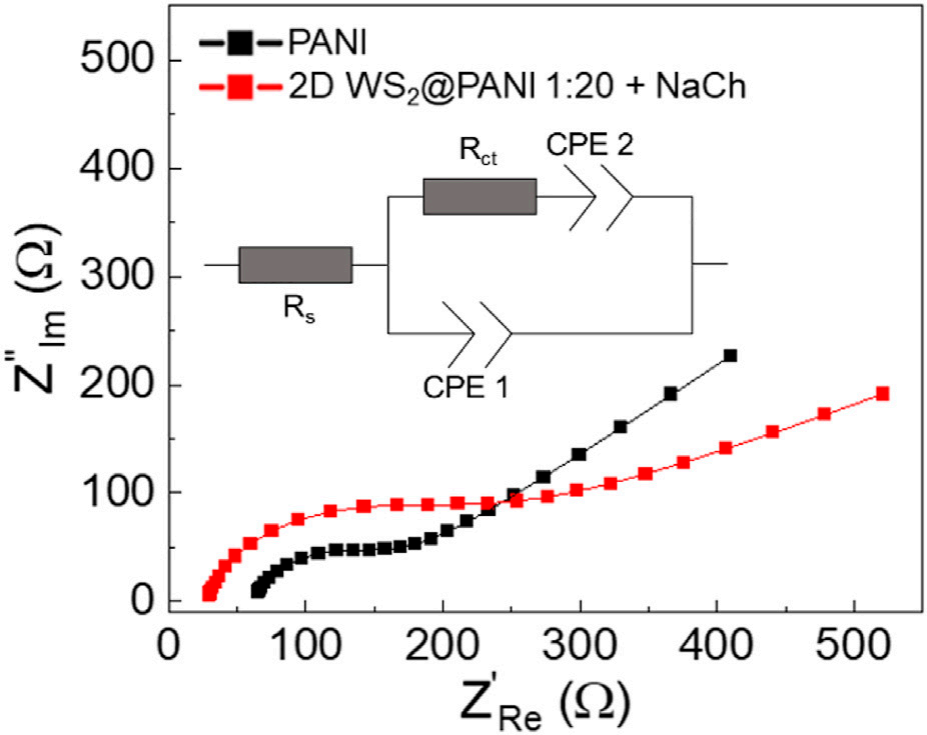
EIS analysis-derived Nyquist plots with relative equivalent circuit for the NaCh-based 1:20 2D WS2@PANI nanohybrid and for pure PANI as measured after GCD analysis.

Stability is a key issue when dealing with modified electrodes: delamination of the coating may occur during measurements and EIS is a really sensitive tool for this scope (Jorcin et al., 2006). To further try to understand the long-term energy storage properties of the NaCh-based 1:20 2D WS2@PANI nanohybrid (the best performing sample) in comparison with the reference PANI sample, EIS analysis was carried out on the electrode after GCD characterization, working in the same electrolyte medium, with the relative Nyquist plots reported in Figure 5. Cyclability of the electrodes was indeed an unsuccessful method to understand durability, since it was visually clear that the active materials were progressively detaching from GC during scans. The results of EIS thus provide information about the electrode-electrolyte interface, i.e., the kinetics of the charge transfer mechanisms happening between WS2 and PANI, as well as about the electrode surface area. The experimental data are fitted using a Randles modified cell (Nguyen and Breitkopf, 2018), choosing a constant phase element (CPE) for modeling the double layer capacitance and the Warburg element (Lamberti et al., 2013): in this way the non-ideality of the whole system can be modeled emphasizing the role of each constituent in the composite electrode. In particular, Rs refers to the saturated resistance, Rct is the charge transfer resistance associated to the sodium cations discharge in the underlying GC electrode, the CPE1 is the associated double layer capacitance and CPE2 represents the Warburg element related to the semi-infinite linear diffusion of the ions from the electrolyte towards the electrode. Fitted values are reported in Table 3. The associated Cdl values from the CPE1 values are calculated using the equations reported in reference (Bard and Faulkner, 2000).

**TABLE 3:** Fitted values from the EIS characterization of the NaCh-based 1:20 2D WS_2_@PANI nanohybrid and for pure PANI after GCD analysis.

Sample	Rs (Ω)	Rct (Ω)	CPE1 (S)	N1	Cdl (uF)	CPE2 (S)	N2
PANI	63	89	1.49e-6	0.87	0.39	0.00051	0.45
2D WS2@ PANI	29	113	7.79e-7	0.89	0.24	0.00076	0.29

The Rct for pure PANI is slightly smaller than the one of the 2D WS_2_@PANI best sample (113 vs. 89 Ω), reflecting a reduced conductivity for the TMD-modified PANI electrode: this result suggests a slower charge discharge at the modified electrode after prolonged GCD characterization. This result is also justified by the steeper slope of the Nyqvist plot (i.e., smaller Warburg impedance) for the pristine PANI sample: the diffusion of ions towards the working electrode is therefore strongly limited by the presence of WS_2_. Furthermore, the presence of a depressed semicircle in both samples (and therefore the use of a CPE instead of an ideal C) accounts for the inhomogeneity of the surface as supported by SEM analysis (Figure 2): more specifically, one expects to find a quasi-tridimensional surface (Mulder et al., 1990). This is the case of the hybrid sample at least, where a fractal morphology is found. However, the N1 fitted value for the two samples (0.87 for PANI sample and 0.89 for the 2D WS_2_@PANI sample) is almost the same thus corroborating the idea that the surface roughness for the two samples is comparable (as also suggested by BET analysis). The delimitation occurred after intense electrochemical characterization accounts for the decrease of about 40% of the value of the Cdl for the composite electrode (0.39 uF for pristine PANI and 0.24 uF for 2D WS_2_@PANI sample) that reflects a smaller surface area for this sample.

The EIS results as a whole suggest that capacitive measurements are not conservative for the electrodes as they have been here characterized (i.e., by relatively simple deposition on a GC) and in order to obtain durable devices, it will be necessary to develop an optimized process for their preparation. We further proof this issue by carrying out multiple cycles of charge/discharge on two prototypical composites (the 1:20 2D WS_2_@PANI hybrids prepared with both NaCh and SDS) at 1 A/g current density. The results, reported in Supplementary Figure S10 over 100 cycles, show how an almost immediate drop in C_S_ of almost 30% takes place after only 10 cycles, likely due to progressive detachment of the active material from the GC electrode during cycling. In general, anyway, we can observe a slightly higher stability of the NaCh-based composite, confirming the better agglomeration between the components in this active material. The incorporation within a gel matrix of the 2D WS_2_@PANI hybrid material is indeed the next step that we are currently exploring in order to produce stable supercapacitors retaining the capacitive properties found in the current study.

## Conclusion

In this work, we report on a series of electro-active composite materials between LPE WS_2_ nanoflakes and *in-situ* polymerized PANI with large interest for application into the next-generation of low-cost, light-weight energy storage devices. We show how the use of different compatibilizers can strongly influence the level of mixing between the two components and consequently the surface properties, with strong implications into charge accumulation mechanisms. In addition, the concomitant effect of using a simple, inexpensive and easy-to-remove templating agent like NaCl during the synthetic process, allows to tune the nano-structuring of the resulting species, with peculiar morphological features emerging in combination with the compatibilizer, which have likely a large influence on the observed capacitive behaviour.

The combined physico-chemical and electrochemical characterization of these species provides significant hints on the structure-property-function relationships, which will serve for the design of even more active nano-structured energy storage materials. Our future plans include their integration into flexible supercapacitor architectures by previous incorporation into hydrogel scaffolds, targeting the field of wearable technologies, which will likely cover a large portion of the market for electronic products in the up-coming decades.

## Data Availability

The raw data supporting the conclusion of this article will be made available by the authors, without undue reservation.
