# Supplementary material for: Nanostructured 2D WS2@PANI nanohybrids for electrochemical energy storage
Source: Front Chem. 2022 Sep 8;10:1000910. doi: 10.3389/fchem.2022.1000910 (PMC9521733; doi:10.3389/fchem.2022.1000910)
Supplement: Supplementary file 1 [file DataSheet1.pdf]

Supplementary Material for:

Nanostructured 2D WS_2@PANI nanohybrids for electrochemical energy storage

Matteo Crisci,[1,2] Felix Boll,[1,2] Jonas Johannes Pflug,[1] Leonardo Merola,[1,3] Zheming Liu,[4] Jaime Gallego,[1,2] Francesco Lamberti,[3] Teresa Gatti[1,2*]

[1] Institute of Physical Chemistry, Justus Liebig University, Heinrich-Buff-Ring 17, 35392 Giessen, Germany

[2] Center for Materials Research, Justus Liebig University, Heinrich-Buff-Ring 17, 35392 Giessen, Germany

[3] Department of Chemical Sciences, University of Padova, via Marzolo 1, 35131 Padova, Italy

[4] Nanochemistry Department, Istituto Italiano di Tecnologia, via Morego 30, 16163 Genova, Italy

Table of Contents

- Raman spectra of LPE WS_2 pre- and post-filtration and after redispersion (Figure S1)
- TEM images of LPE WS_2 (Figure S2)
- SEM images of template-assisted oxidatively polymerized pure PANI, of a 2D WS_2@PANI nanohybrid prepared without any surfactant and of LPE WS_2 casted from the colloidal phase onto a substrate (Figure S3)
- SEM images of 2D WS2@PANI nanohybrids obtained with different LPE WS_2/PANI molar ratios in the presence of NaCh (Figure S4)
- SEM images of 2D WS2@PANI nanohybrids obtained with different LPE WS_2/PANI molar ratios in the presence of SDS (Figure S5)
- N_2 (77 K) physisorption isotherm of a prototypical 2D WS_2@PANI nanohybrid and pore size distribution calculated from the adsorption and desorption branches using the NLDFT method (Figure S6)
- Full XPS spectra of 1:20 2D WS_2@PANI nanohybrids prepared in the presence of SDS and NaCh (Figure S7)
- EDX maps of different elements in 1:20 2D WS_2@PANI nanohybrids prepared in the presence of SDS and NaCh (Figure S8)
- GCD curves at increasing current densities for the 2D WS2@PANI nanohybrids (Figure S9)
- Cyclability of the 1:20 2D WS_2@PANI nanohybrids prepared in the presence of SDS and NaCh measured at a current density of 1 A/g over 100 cycles (Figure S10)

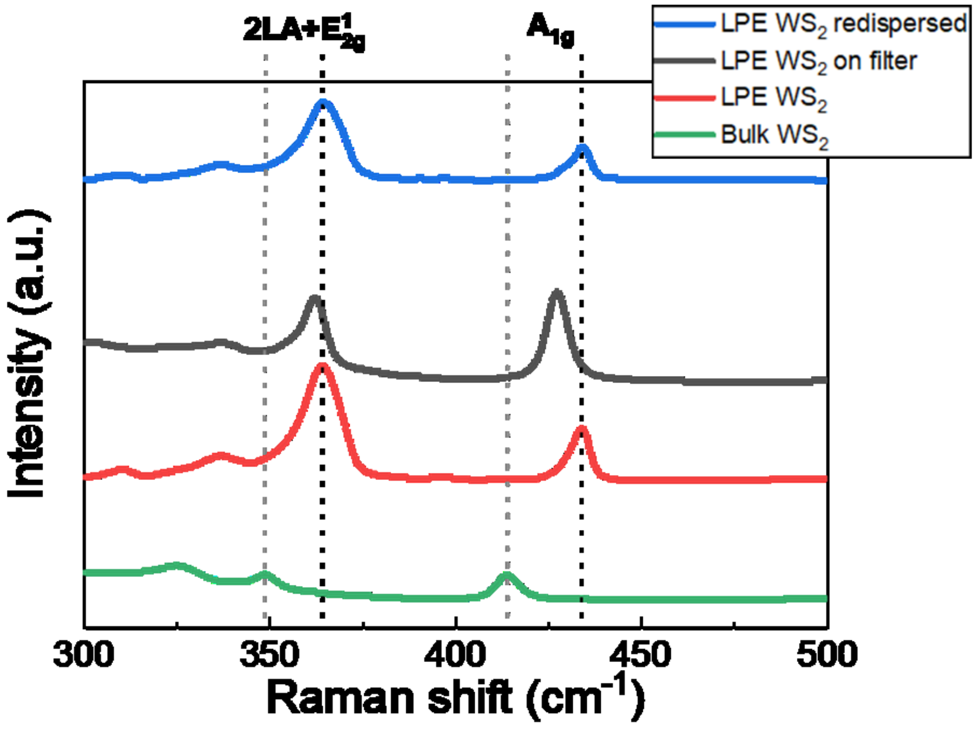

Figure S1. Raman spectra LPE WS_2 pre- and post-filtration and after redispersion of the filtrated material in 1 HCL in brine in the presence of a freshly added surfactant. The spectrum of bulk WS_2 powder is also shown for the sake of comparison.

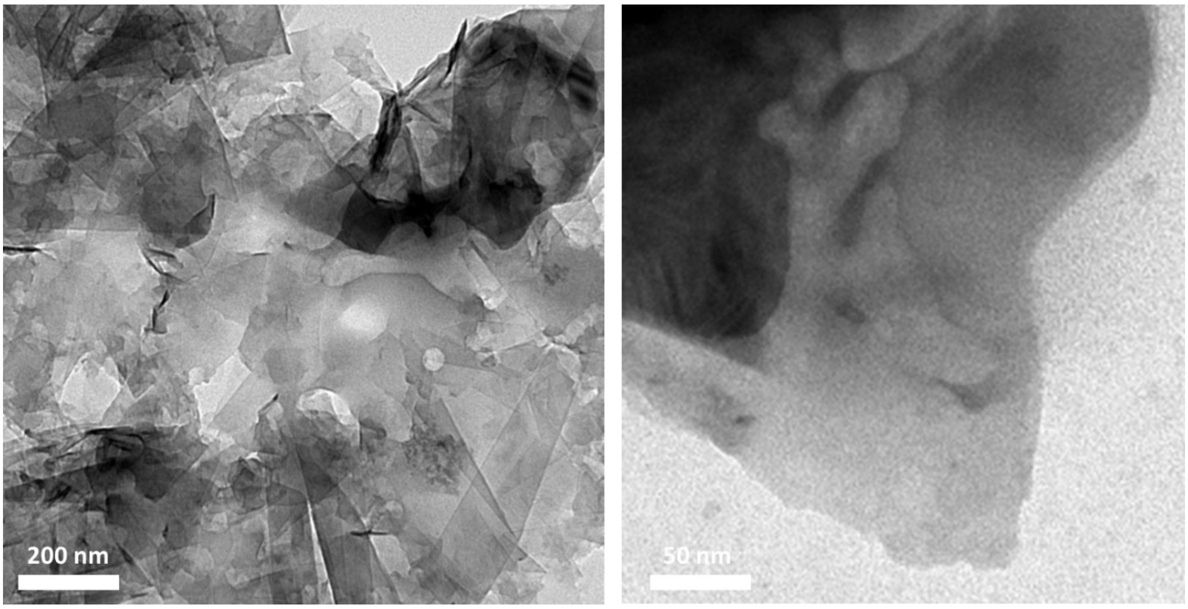

Figure S2. TEM images of LPE WS_2.

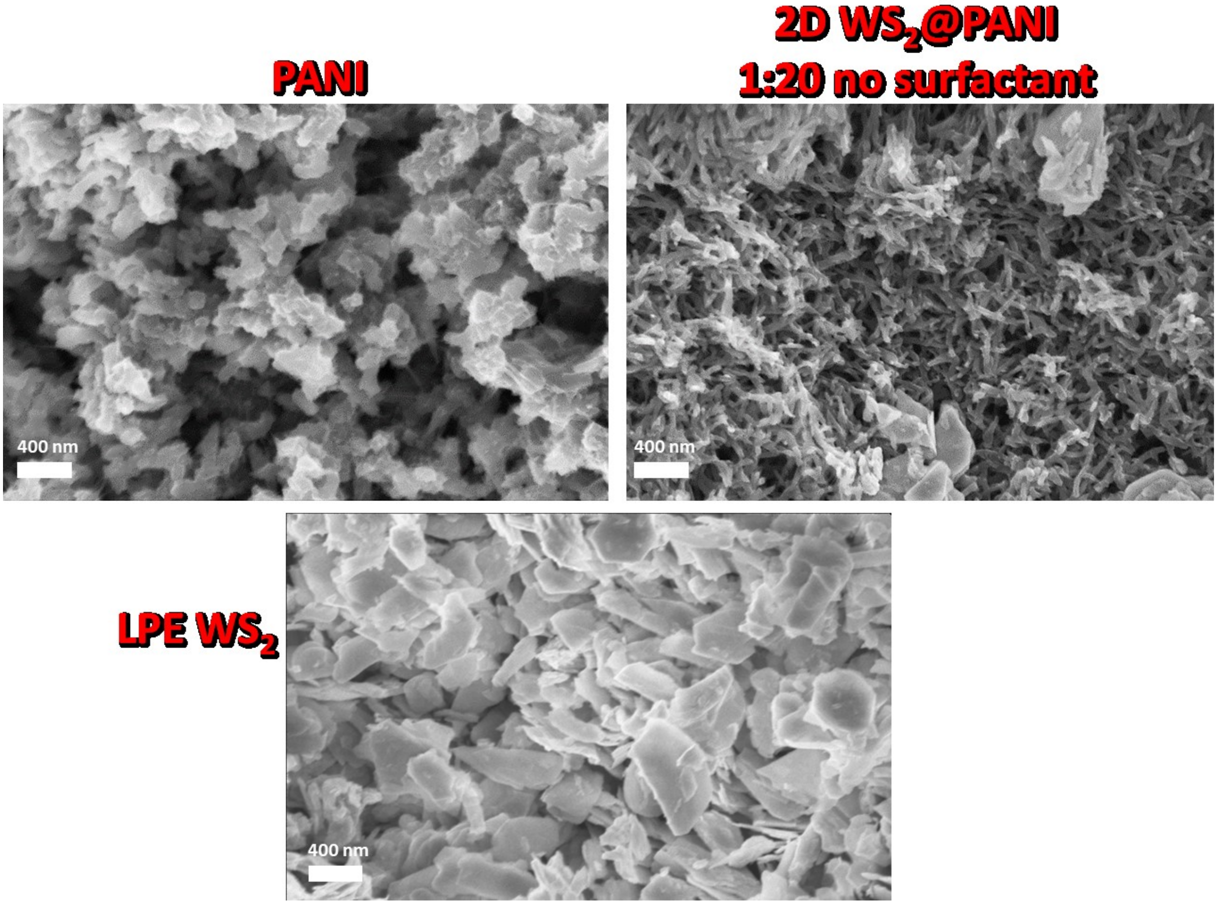

Figure S3. SEM images of template-assisted oxidatively polymerized pure PANI, of a 2D WS2@PANI nanohybrid prepared without the addition of any surfactant and of LPE WS_2 casted from the colloidal phase onto a substrate.

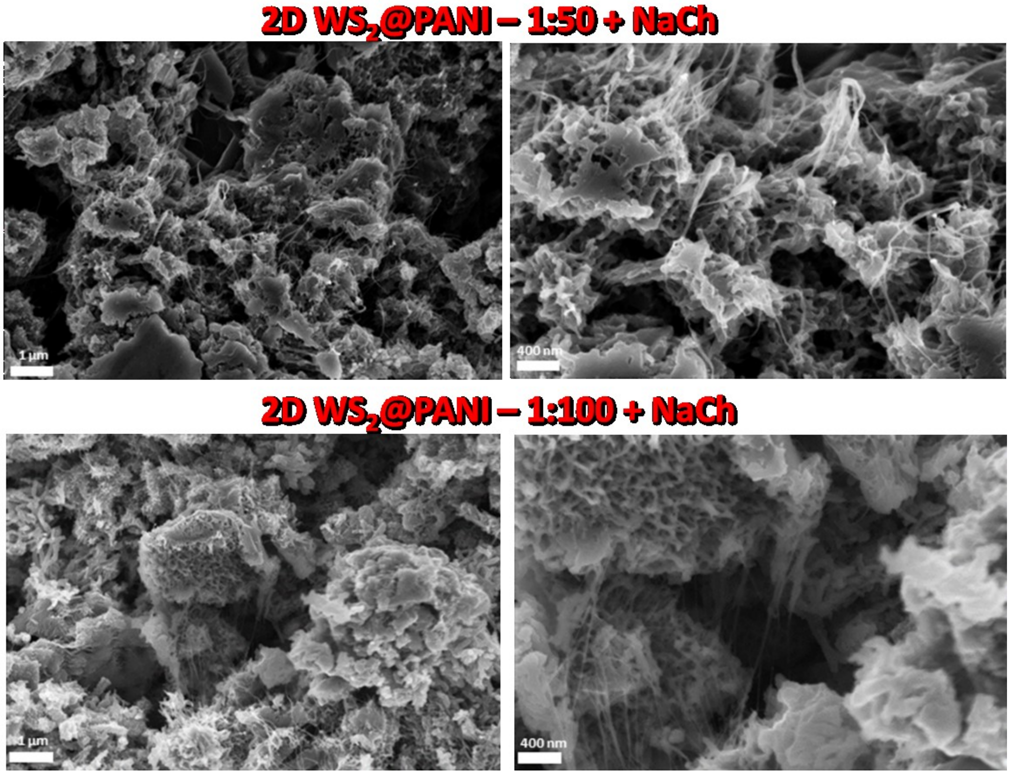

Figure S4. SEM images of 2D WS2@PANI nanohybrids obtained with different LPE WS_2/PANI molar ratios in the presence of NaCh.

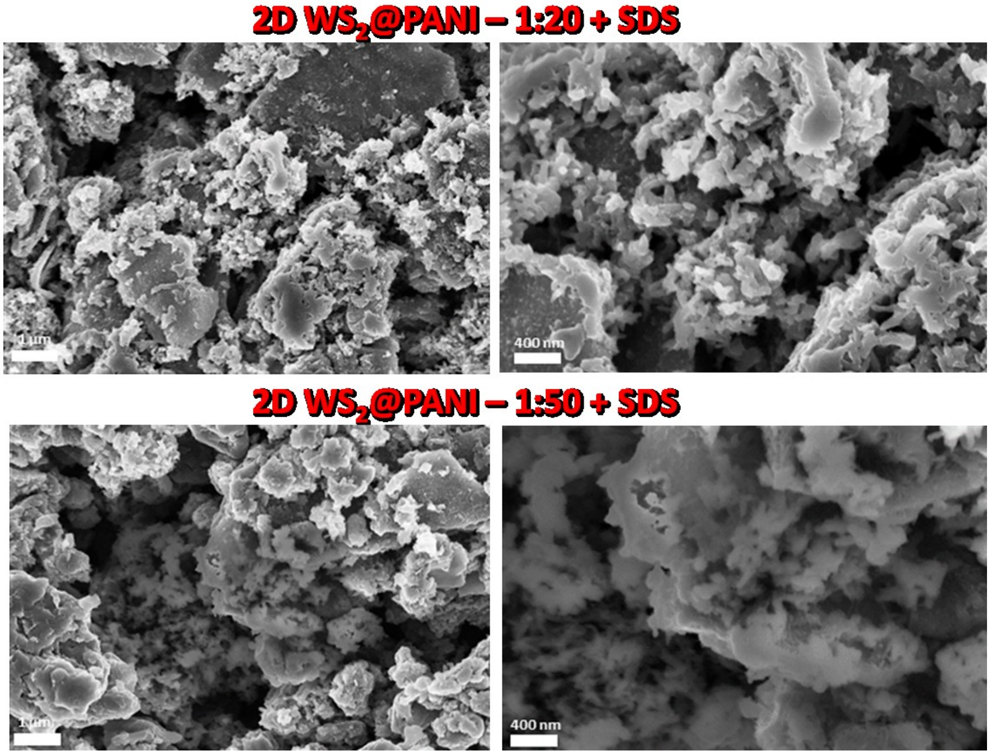

Figure S5. SEM images of 2D WS2@PANI nanohybrids obtained with different LPE WS_2/PANI molar ratios in the presence of SDS.

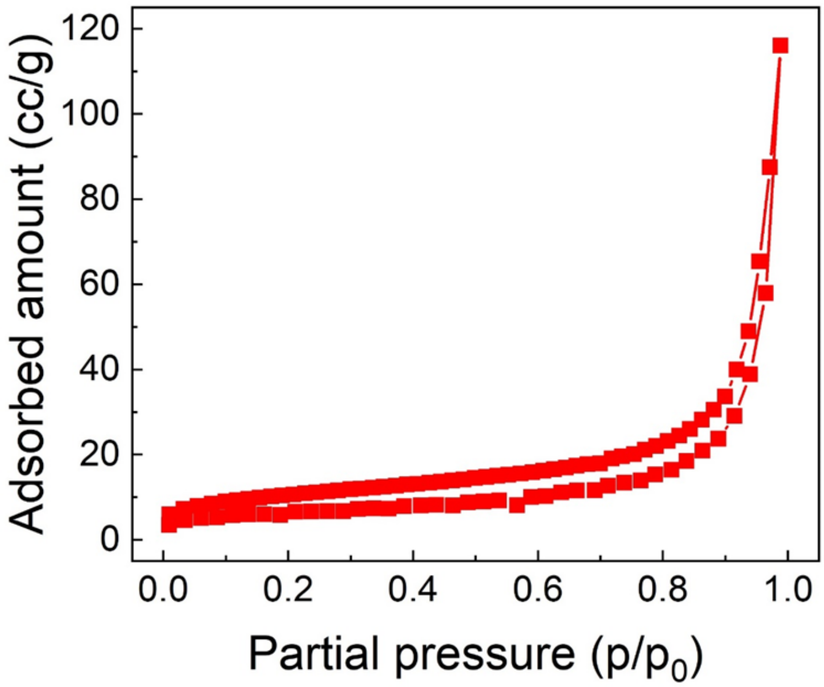

Figure S6. a) N_2 (77 K) physisorption isotherm of a prototypical 2D WS2@PANI nanohybrid. The total surface area is calculated from the adsorption and desorption branches using the NLDFT method.

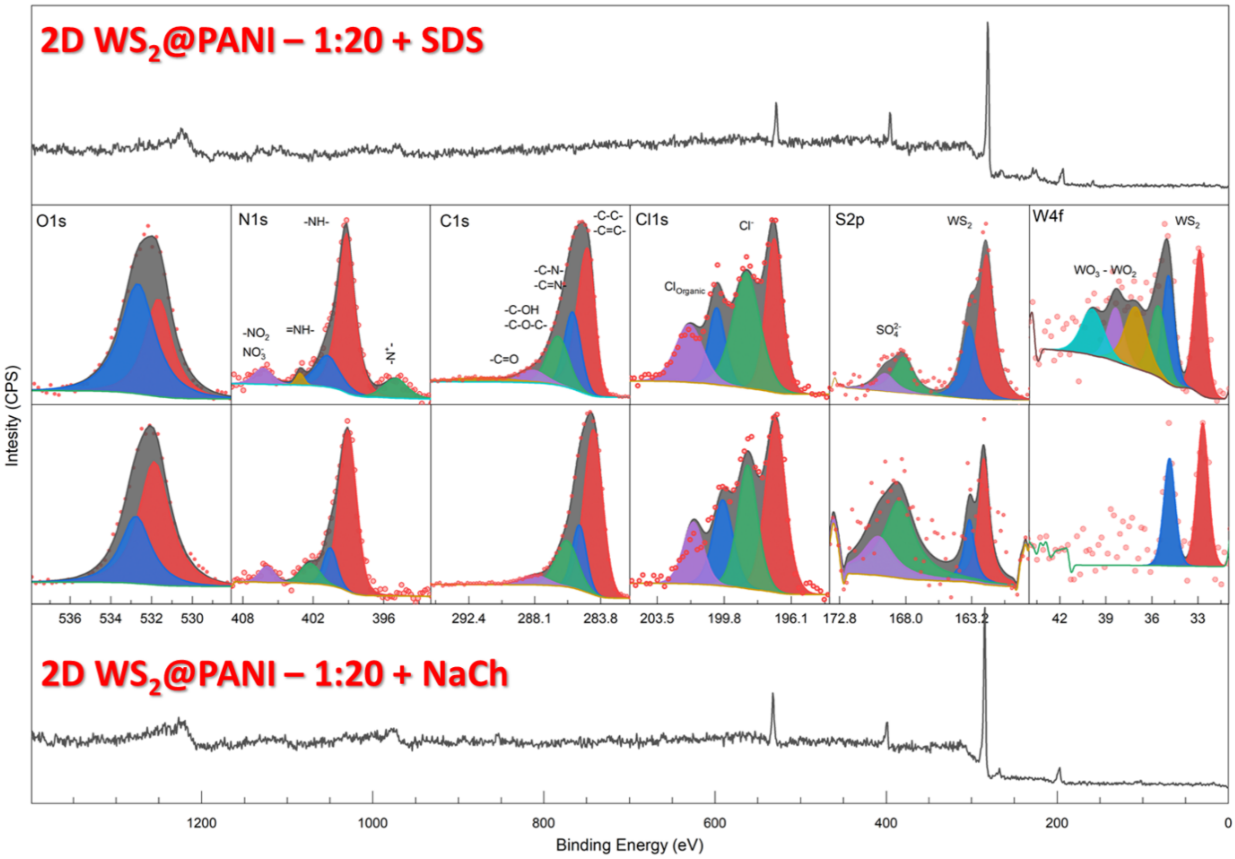

Figure S7. Full XPS spectra of 1:20 2D WS2@PANI nanohybrids prepared in the presence of SDS and NaCh.

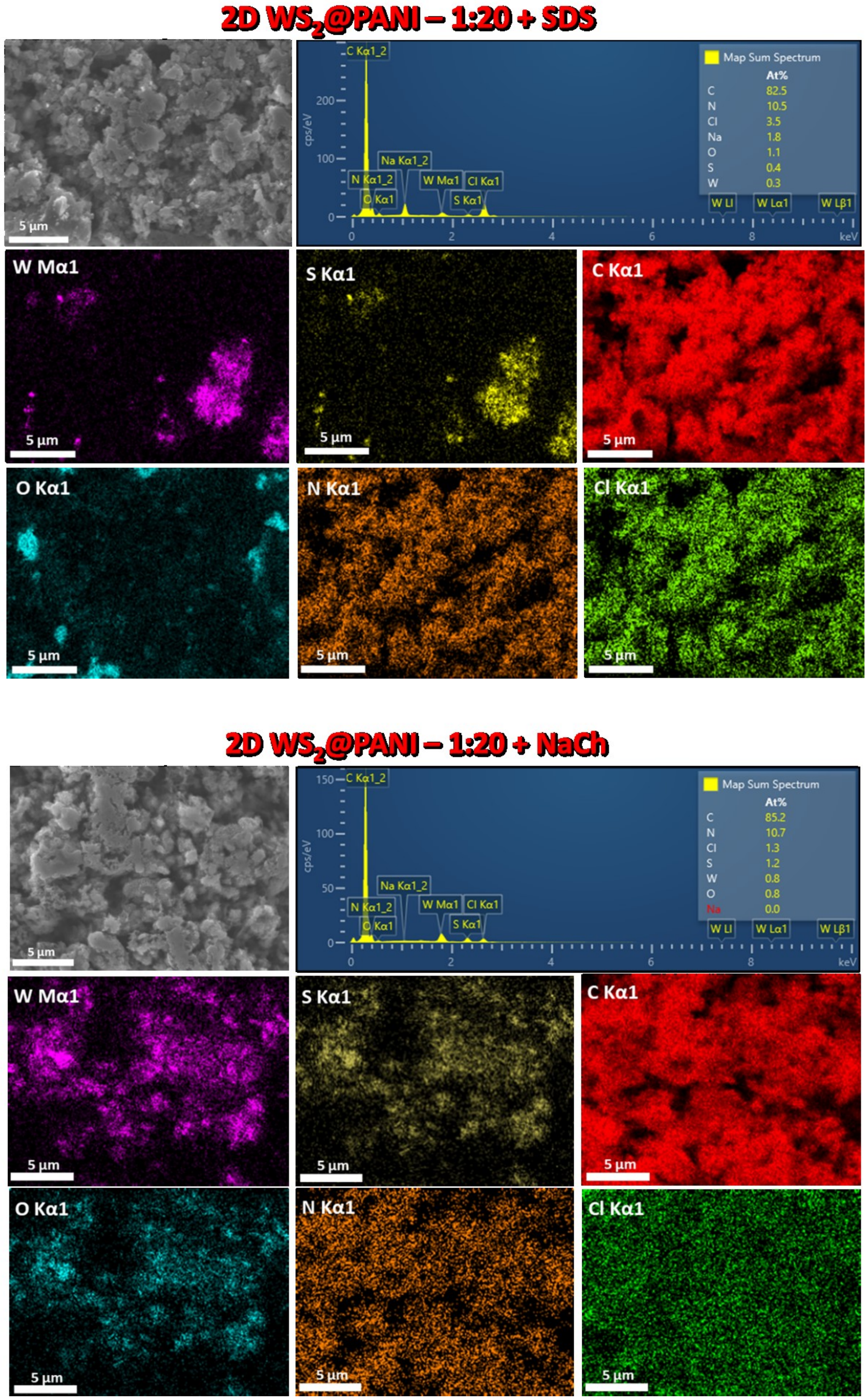

Figure S8. EDX maps of different elements in 1:20 2D WS_2@PANI nanohybrids prepared in the presence of SDS and NaCh.

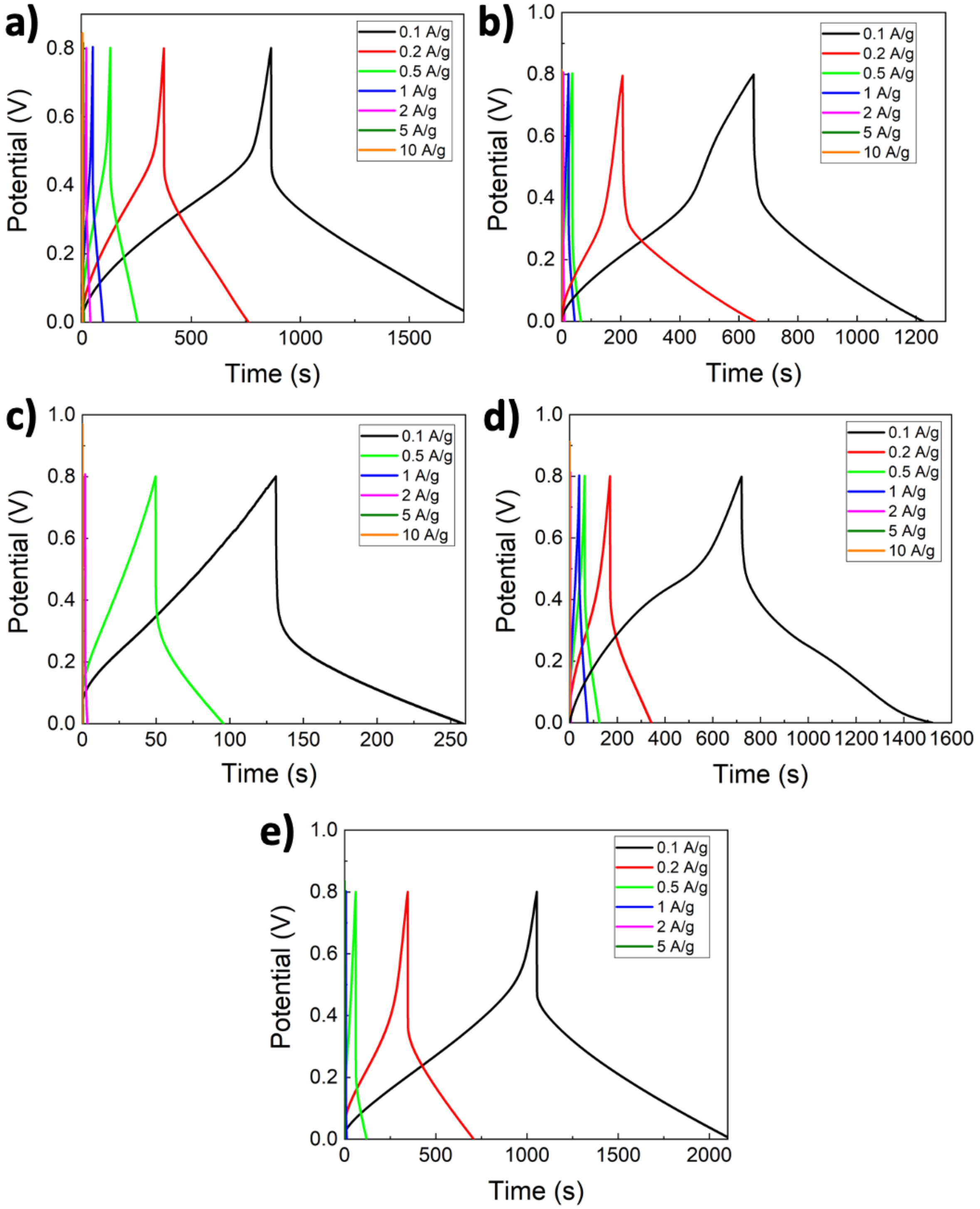

Figure S9. GCD curves at increasing current densities for different 2D WS2@PANI nanohybrids: a) 1:50 + NaCh, b) 1:100 + NaCh, c) 1:20 + SDS, d) 1:50 + SDS, e) 1:100 + SDS.

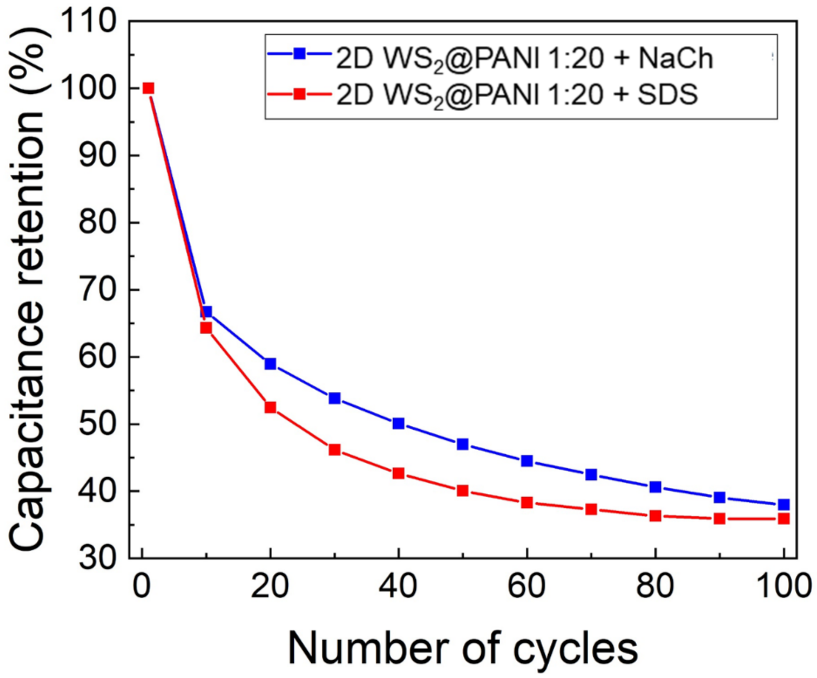

Figure S10. Cyclability of the 1:20 2D WS_2@PANI nanohybrids prepared in the presence of SDS and NaCh measured at a current density of 1 A/g over 100 cycles in a three electrode system (with the active materials deposited on the GC electrode).
